# Marinicellulosiphila megalodicopiae gen. nov., sp. nov., a deep-sea alkaliphilic cellulolytic bacterium isolated from an endemic ascidian Megalodicopia hians

**DOI:** 10.1099/ijsem.0.006742

**Published:** 2025-04-02

**Authors:** Mikako Tachioka, Masayuki Miyazaki, Mikiko Tsudome, Miwako Tsuda, Kohsuke Uchimura, Yoshihiro Takaki, Shigeru Deguchi

**Affiliations:** 1 Research Center for Bioscience and Nanoscience, Japan Agency for Marine-Earth Science and Technology (JAMSTEC), 2-15 Natsushima-cho, Yokosuka 237-0061, Japan; 2 Super-Cutting-Edge Grand and Advanced Research (SUGAR) Program, JAMSTEC, 2-15 Natsushima-cho, Yokosuka 237-0061, Japan; 3 Project Team for Development of New-Generation Research Protocol for Submarine Resources, JAMSTEC, 2-15 Natsushima-cho, Yokosuka 237-0061, Japan

**Keywords:** cellulose degradation, deep-sea, *Gynuellaceae*, *Oceanospirillales*, *Pseudomonadales*, *Saccharospirillaceae*

## Abstract

The strain TOYAMA8^T^ is a deep-sea alkaliphilic cellulolytic bacterium isolated from a slurry-adhered epiphytic site of *Megalodicopia hians*. Cells of this strain are Gram-negative, aerobic, curved rods or spirilla, motile with monopolar flagella, and grow on cellulose as the sole carbon source. Compared to other closely related species, this bacterium is characterized by a large number of cellulase genes. Strain TOYAMA8^T^ showed alkaliphilic growth within the pH range 7.5–9.0. The major cellular fatty acids were C_18 : 1_ ω7, C_14 : 0_, C_16 : 0_ and C_16 : 1_ ω7. The major polar lipids were phosphatidylglycerol, phosphatidylethanolamine, unidentified phospholipids and aminolipids. A major respiratory lipoquinone was Q-9. Phylogenomic analysis using the 16S rRNA gene and whole-genome sequence data showed that the strain is related to the families *Gynuellaceae*, *Saccharospirillaceae* and *Natronospirillaceae*. The values of 16S rRNA gene sequence similarity, amino acid identity and percentage of conserved proteins between the strain TOYAMA8^T^ and related species were low, with maximum values of 90.6, 48.1 and 34.6%, respectively. These results, together with differences in phenotypic and biochemical characteristics, indicate that the new isolate TOYAMA8^T^ represents a novel genus and species, for which the name *Marinicellulosiphila megalodicopiae* gen. nov., sp. nov., is proposed. The type strain is TOYAMA8^T^ (JCM 31119^T^=DSM 114864^T^).

Cellulose is a linear homopolymer of *β*-1,4-linked glucose that is produced primarily as a structural polysaccharide in plant cell walls, but also in algae, tunicates and certain bacteria. As cellulose is the most abundant organic matter on Earth, microbial degradation and utilization are essential for the carbon cycle. Microbial enzymes capable of degrading recalcitrant cellulose are critical for establishing a sustainable society, as they produce chemicals from biomass feedstock instead of fossil fuels.

Although most photosynthetic cellulose production occurs in terrestrial ecosystems, the deep sea, a large part of the ocean that covers 71% of the Earth’s surface, also harbours cellulose. In these aphotic zones, organic matter derived from surface waters sinks to depths where easily digestible components are rapidly consumed, leaving behind recalcitrant materials such as cellulose [1]. Therefore, the ability to degrade cellulose may be a key strategy for the survival of deep-sea micro-organisms. However, cellulolytic activity in the deep sea remains understudied, partly because of the challenges in detecting and culturing these specialized microbes under extreme or unestablished conditions.

A recently developed nanofibre-based assay has enabled the ultrasensitive detection of cellulase activity at the nanoscale and has revealed that a diverse range of cellulose-degrading bacteria also inhabit the deep sea and play an active role in cellulose degradation [2,3]. Several deep-sea bacterial strains have shown robust cellulolytic activity [2], including *Marinagarivorans cellulosilyticus* strain GE09 [4]. Another intriguing example is strain TOYAMA8, which was isolated from the deep-sea ascidian *Megalodicopia hians* in Toyama Bay, Japan.

*M. hians* has been found in the bathyal/abyssal zone [5] and is notable for its large mouth-like siphons. Tunicates produce cellulose as a protective coat or food-trapping filter [6], and because tunicate cellulose synthesis does not involve photosynthesis, they represent an exceptional source of cellulose in the deep sea, making them a unique focus for the isolation of cellulolytic microbes. Genomic and transcriptomic analyses of strain TOYAMA8 revealed that it utilizes a distinct set of enzymes compared to known terrestrial cellulases [7]. These findings suggest that deep-sea bacteria evolved unique enzyme production strategies to adapt to nutrient-limited conditions. Exploring these adaptations could reveal novel biochemical mechanisms that enable survival in extreme deep-sea environments and pave the way for the discovery of new enzymes and biocatalytic processes.

The order *Oceanospirillales* is a major group of *Gammaproteobacteria* [8]. *Saccharospirillaceae* is a member of *Oceanospirillales* and was described by Kevbrin *et al*. [9] with a revision of the tentative proposal for the family name in 2005 [10]. In the current classification, the family *Saccharospirillaceae* includes the genera *Reinekia*, *Saccharospirillum* and *Salinibius* (https://lpsn.dsmz.de/family/saccharospirillaceae). The closely related genera *Natronospirillum*, *Salinispirillum* and *Gynuella*, which were previously classified in the provisional family ‘*Saccharospirillaceae*’, form the families *Natronospirillaceae* and *Gynuellaceae* [9]. A recent multigene-based phylogenomic tree approach has also led to the reclassification of the order *Oceanospirillales*, resulting in its merger with the order *Pseudomonadales* [8], consistent with the Genome Taxonomy Database (GTDB) classification [11].

Here, we report the phylogenetic and phenotypic characterization of a new isolate, TOYAMA8, obtained using a highly sensitive technique to detect the cellulolytic activity of microorganisms [2,3].

## Isolation

*M. hians* has been found in Toyama Bay, Japan, at depths of 592–978 m [5]. In this study, an individual *M. hians*, collected by local fishermen in their fishing nets in Toyama Bay, Japan (37.08 N 137.15 E), with a water depth of >800 m, was employed as a source for isolating cellulose degraders using the surface pitting observation technology [2,3]. Cellulolytic bacteria could not be isolated from the surface of the specimens. The cellulolytic strain TOYAMA8 was isolated from deep-sea sediments attached to a specimen. Briefly, a nanofibrous cellulose plate was prepared as described previously [12,13], with an exchange of media containing artificial seawater (ASW, Marine Art SF-1; Senju Pharmaceutical), 1 mM ammonium sulphate and trace minerals (solution A from *Teredinibacter* medium, ATCC medium #1983). Visible pits that appeared on the surface of the inoculated cellulose plate after 7 days of incubation at 20 °C were used as indicators for the initial isolation of cellulolytic bacteria [2]. Further isolation was performed using fresh cellulose or marine agar (MA) 2216 (Difco) plates until a pure culture was obtained. The bacteria were stored at –80 °C in 20% (v/v) glycerol.

## 16S rRNA phylogeny and genome features

The strain TOYAMA8 was cultivated in liquid culture containing ASW, 1 mM ammonium sulphate, trace minerals, 0.2% cellobiose and 0.1% casamino acids at 20 °C for 4 days. The cells were collected by centrifugation, and the genome was extracted using a MagAttract HMW DNA kit (Qiagen) according to the manufacturer’s instructions, with slight modifications. The extracted DNA was sequenced using a PacBio Sequel at DNA Link (Seoul, Korea). An Illumina paired-end library was constructed from the extracted DNA using the KAPA Hyper Prep Kit Illumina platform (KAPA Biosystems, Woburn, MA, USA) according to the manufacturer’s instructions and sequenced on an Illumina MiSeq platform with 300 bp paired-end sequences. Raw reads were processed using Trimmomatic v0.36 [14] to trim the remaining adaptor and low-quality sequences. *De novo* assembly was performed using the HGAP4 [15] pipeline of the PacBio SMRT Toolkit (SMRT Link v6.0.0).

Multiple alignments of the 16S rRNA genes were performed using the muscle program [16], and gaps were trimmed using TrimAl v1.2 [17] to a total of 1,266 bp in the final dataset. Phylogenetic and molecular evolutionary analyses were conducted using the mega program package v11 [18] as follows: phylogenetic tree reconstruction and bootstrap resampling analysis were performed using maximum-likelihood, neighbour-joining and maximum-parsimony methods. Pairwise sequence similarity values were calculated using blast (https://blast.ncbi.nlm.nih.gov/). A concatenated alignment of 120 conserved single-copy protein-coding marker genes was prepared using GTDB-tk v1.7 [19], and gaps were trimmed using TrimAl v1.2 [17] to a total of 4,020 aa in the final dataset. A maximum-likelihood tree was constructed using the mega program package v11 [18]. The average nucleotide identity (ANI) was calculated using the OrthoANI calculator [20]. The average amino acid identity (AAI) was calculated using an online AAI calculator from Kostas Lab [21]. The percentage of conserved proteins (POCP) was calculated using a Bash script (https://doi.org/10.6084/m9.figshare.4577953.v1) [22] based on the methods described by Qin *et al*. [23]. To analyse carbohydrate-active enzyme (CAZyme) genes, the dbCAN3 server [24] was used to search for CAZyme domains and signal peptides. Annotations were accepted only if at least two of the three tools in dbCAN3 yielded positive results.

**Fig. 1.**
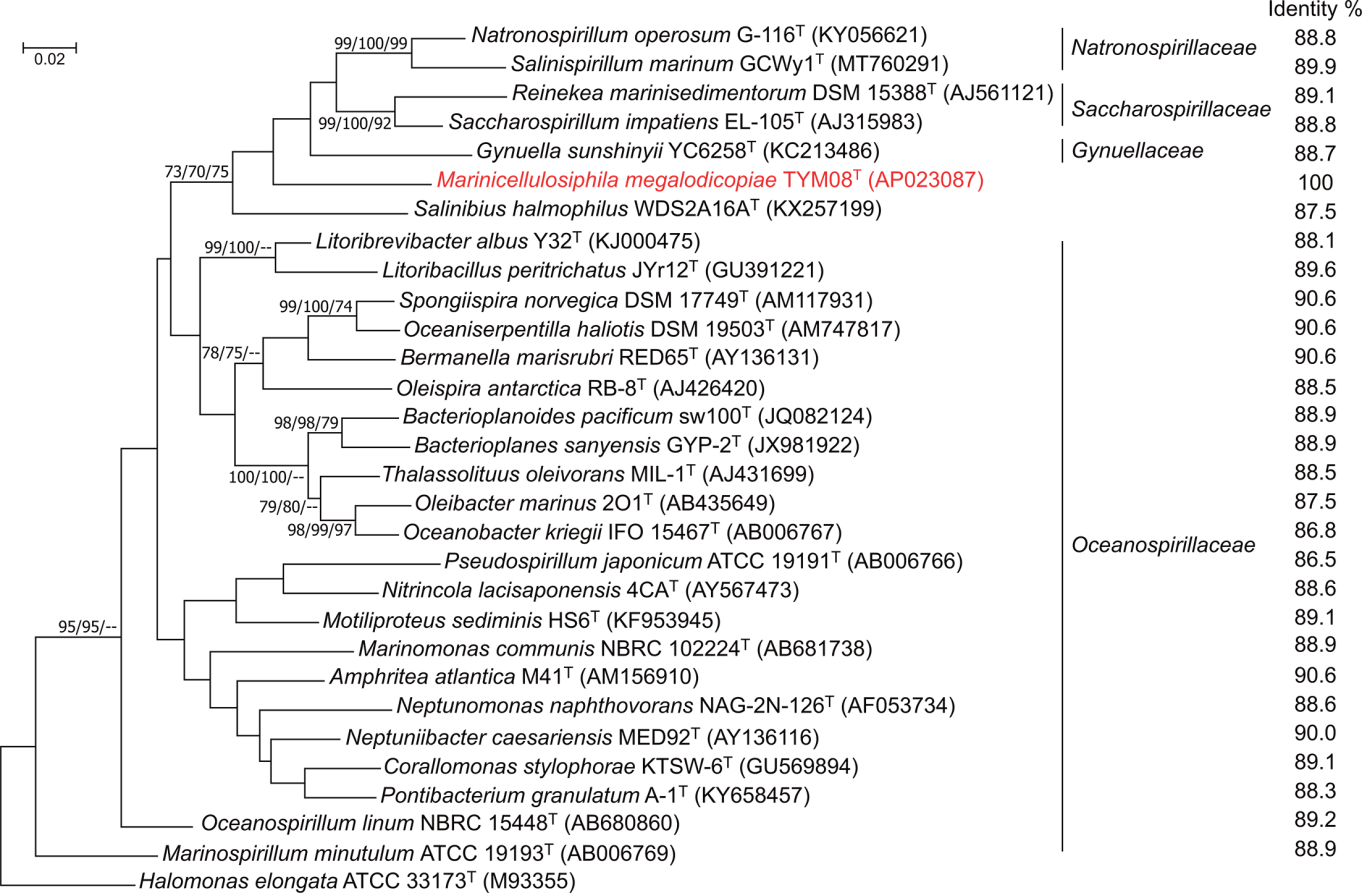
Maximum-likelihood phylogenetic tree based on 16S rRNA gene sequences of the strains in the families *Gynuellaceae*, *Saccharospirillaceae*, *Natronospirillaceae* and *Oceanospirillaceae* of the order *Oceanospirillales*. Numbers at the nodes are bootstrap percentages based on the maximum-likelihood/neighbour-joining/maximum-parsimony methods derived from 1,000 replications; only values ≥70% are shown. GenBank accession numbers of 16S rRNA gene sequences are shown in parentheses. The tree was rooted using *Halomonas elongata* ATCC 33173^T^ as the outgroup. Bar, 0.02 substitutions per nt position.

Six copies of the 16S rRNA genes were found in the TOYAMA8 genome, in which the difference was observed at ten positions in the total length of 1,536 bp, and each sequence shared 99.3–100% identities. The strain TOYAMA8 shared a maximum of ~91% 16S rRNA gene sequence identity with members of the family *Oceanospirillaceae* containing the genera *Amphritea*, *Bermanella*, *Oceaniserpentilla* and *Spongiispira* (Fig. 1). Although the phylogenetic relationships were uncertain, as indicated by low bootstrap support, all phylogenetic trees based on the maximum-likelihood, neighbour-joining and maximum-parsimony methods placed the strain TOYAMA8 in a clade associated with the genera *Salinibius* and *Gynuella* and the families *Saccharospirillaceae* and *Natronospirillaceae*. Originally, these genera belonged to a provisional family ‘*Saccharospirillaceae*’, which was subsequently split into *Gynuellaceae*, *Saccharospirillaceae* and *Natronospirillaceae* by Kevbrin *et al*. [9]. However, another study in 2020 proposed *Salinibius* as a new genus that retained the provisional family designation [25], leading to *Salinibius halmophilus* being unintentionally classified under *Saccharospirillaceae* (https://lpsn.dsmz.de/family/saccharospirillaceae). The sequence identity values between the strain TOYAMA8 and other members of the families *Gynuellaceae*, *Saccharospirillaceae* and *Natronospirillaceae* were 87.5–89.1%.

**Fig. 2.**
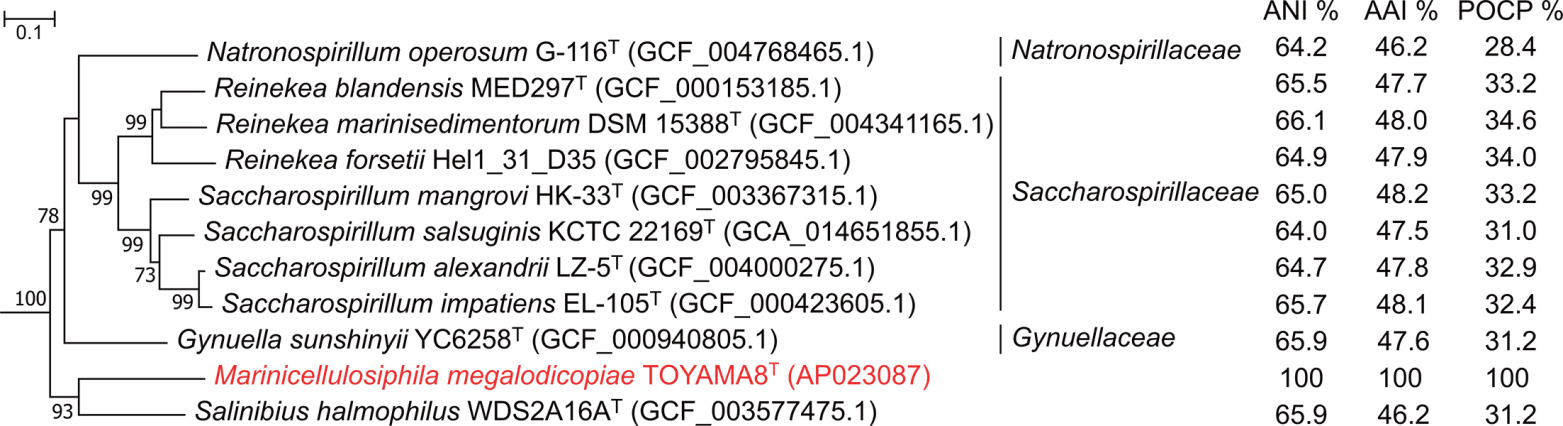
Maximum-likelihood tree based on 120 concatenated single-copy marker proteins of the strain TOYAMA8 and members in the families *Gynuellaceae*, *Saccharospirillaceae* and *Natronospirillaceae*. Bootstrap values (≥70%, 300 replications) are shown at the nodes. The figure presented here is a subtree extracted from the larger tree shown in Fig. S1. Bar, 0.1 substitutions per nt position.

Complete genome sequencing of the strain TOYAMA8 yielded a single circular chromosome of 4,648,065 bp containing 3,906 protein-coding sequences. The genome size was intermediate compared with that of other related species (Table 1). The G+C content of strain TOYAMA8 (36.1 mol%) was notably lower than that of other related species (45 mol%; Table 1). Genome-based phylogenetic analysis using the GTDB-tk tool also supported the grouping of strain TOYAMA8, along with *S. halmophilus*, *Gynuella sunshinyii* and the species in the families *Saccharospirillaceae* and *Natronospirillaceae* (Figs 2 and S1, available in the online Supplementary Material). The pairwise AAI, POCP and ANI values between the strain TOYAMA8 and other closely related species were <50, ~30 and ~65%, respectively (Fig. 2). Because the general thresholds for genus delineation are AAI of 60–65% and POCP of 50% [26], our estimates met the criteria for proposing a new genus. The low 16S rRNA gene sequence identity, AAI and POCP values were close to the family-level separation threshold [9]. Our phylogenetic analyses suggest that the inclusion of *S. halmophilus* in the family *Saccharospirillaceae* leads to nomenclatural conflict, which arises from the fact that proposals for the genus *Salinibius* and the family *Gynuellaceae* were published in the same year [9,25]. To address this issue and avoid placing our isolate within an erroneous taxonomic framework, we propose the inclusion of *Marinicellulosiphila* gen. nov. and *Salinibius* as *incertae sedis* in the order *Oceanospirillales* (or *Pseudomonadales*), pending further evidence to clarify its correct family assignment.

**Table 1.** Differential characteristics between TOYAMA8 and related species Taxa: 1, strain TOYAMA8^T^; 2, *S. halmophilus* WDS2A16A^T^; 3, *G. sunshinyii* YC6258^T^; 4, *Reinekea* spp.; 5, *Saccharospirillum* spp.; 6, *Salinispirillum marinum* GCWy1^T^; 7, *Natronospirillum operosum* G-116^T^. We used the data from previous studies (Ling *et al*. [25], Chung *et al*. [28], Kang *et al*. [38], Choi and Cho [37], Pinhassi *et al*. [35], Romanenko *et al.* [36], Kim *et al*. [39], Labrenz *et al*. [40], Zhang *et al*. [41], Chen *et al*. [42], Choi *et al*. [43], Fidalgo *et al*. [44], Yang *et al*. [45], Shahinpei *et al*. [46] and Kevbrin *et al*. [9]). +, positive; −, negative; w, weak; NR, not reported.

	1	2	3	4	5	6	7
Source	Sea squirt(*M. hians*)	Marine solar saltern in Weihai, PR China	Tidal flat sediment	Seawater, surface seawater and tidal flat sediment	Hypersaline lake, salt mine, tidal flat sediment, halophyte and marine dinoflagellate	Coastal marine	Cyanobacterium
Morphology	Curved rods or spirilla	Spirilla	Rods	Curved, straight or slightly bent rods	Curved rods or spirilla	Spirilla	Spirilla
Size (μm)	0.3–0.5×1.8–5.4	0.4–0.6×3.0–8.0	1.5–1.8×2.8–3.1	0.2–0.7×1.2–4.0	0.3–1.0×2.0–12.0	0.3–0.5×2.5–5.9	0.3–0.4×5.0–11.0
Motility	Motile	Motile	Motile	Non-motile or motile	Non-motile or motile	Motile	Motile
Growth response to O_2_*	OA	FA	FA	OA, FA	OA, MA, FA	FA	OA
Optimal temp.	20	33–37	25–35	25–30	16–40	30	31–37
Temp. range	5–26	20–40	10–40	4–42	<2.5–50	4–40	4–45
Optimal NaCl (%)	1.5–2.5	3–4	1–4	2–5	2–6	3	3.5–6
NaCl range (%)	0.5–5.5	1–15	1–7	0.3–12	<1–15	1.0–10	1.0–20
pH range	7.5–9	6–9	4.5–9.5	5–12	5–12	7.5–10	7.3–10
Major respiratory quinone	Q-9	Q-8	Q-8	Q-8	Q-8	Q-9	Q-8
Polar lipids†	PG, PE, PL, AL	PG, DPG, GL, PE, AGL	DPG, PG, PE, UL	PG, PE, DPG, PI, PS, PL, AL, UL	DPG, PG, PE, LPE, MMPE, APGL, PL, PC, GL, UL	DPG, PG, PE, AL, UL	PE, PG, DPG, PL, AL, UL
DNA G+C contents (mol%)§	36.1(WGS)	48.5(WGS)	48.9(HPLC)	45.5–52.4 (HPLC/WGS)	54.5–57.3 (HPLC/WGS)	52.3(HPLC)	60.4(T_m_)
Genome size (Mbp)	4.6	3.7	6.5	2.9–4.5	3.7–5.5	NR	4.3
Major fatty acids‡	C_14 : 0_, C_18 : 1_ ω7	C_16 : 0_, C_18 : 1_ ω7c	C_16 : 0_, C_18 : 1_ ω7c, summed feature 2, summed feature 3	C_16 : 0_, summed feature 3, summed feature 8	C_16 : 0_, summed feature 3, summed feature 8	C_18 : 1_ ω7c, C_16 : 0_	C_16 : 0_, summed feature 3, summed feature 8, C_19 : 0_ cyclo ω8c
Hydrolysis of:							
Cellulose	+	−¶	+	+, −¶	+, −¶	NR	−¶
Aesculin	+	NR	+	+	+, −	−	−
Gelatin	−	+	+	−	+, −	+	−
Urea	+	−	−	−	−	−	−
Growth on:							
ᴅ-Xylose	−	−	+	−**	+††, −	−	+
Mannose	−	NR	+	+**	+, −	NR	−
ᴅ-Fructose	−	+	+	+, −	+, −	NR	+
Sucrose, maltose	−	+	+	+, −	+, −	+	+
API ZYM test:							
Esterase (C4)	+	−	+	+, −	+, −	NR	+
Esterase lipase (C8)	+	−	−	w, −	+	NR	+
Lipase (C14)	−	−	+	−	−	NR	−
Valine arylamidase	+	−	+	−	+**	NR	−
*α*-Glucosidase	−	+	+	+, −	+	NR	+
ß-Glucosidase	+	−	+	+, −	+, −	NR	+

*OA, obligate aerobe; MA, microaerophilic; FA, facultative anaerobe

†PG, phosphatidylglycerol; DPG, diphosphatidylglycerol; PE, phosphatidylethanolamine; LPE, lyso-phosphatidylethanolamine; MMPE, monomethyl-phosphatidylethanolamine; PI, phosphatidylinositol; PC, phosphatidylcholine; APGL, aminophosphoglycolipid; PLs, unidentified phospholipids; GLs, unidentified glycolipids; ALs, aminolipids; ULs, unidentified lipids.

‡Summed Ffeatures are fatty acids that cannot be resolved reliably from another fatty acid using the chromatographic conditions chosen. The MIDI system groups these fatty acids together as a single feature, with a single percentage of the total. Summed feature 2 comprised C_12 : 0_ aldehyde, summed feature 3 comprised C_16 : 1_ ω6c and/or C_16 : 1_ ω7c, and summed feature 8 comprised C_18 : 1_ ω6c and/or C_18 : 1_ ω7c.

§Determined by whole-genome sequencing (WGS), high-performance liquid chromatography (HPLC), or melting temperature (T_m_).

a;¶Activity determined using carboxymethyl cellulose.

b;**Not reported for all strains.

c;††Tested by acid production.

**Fig. 3.**
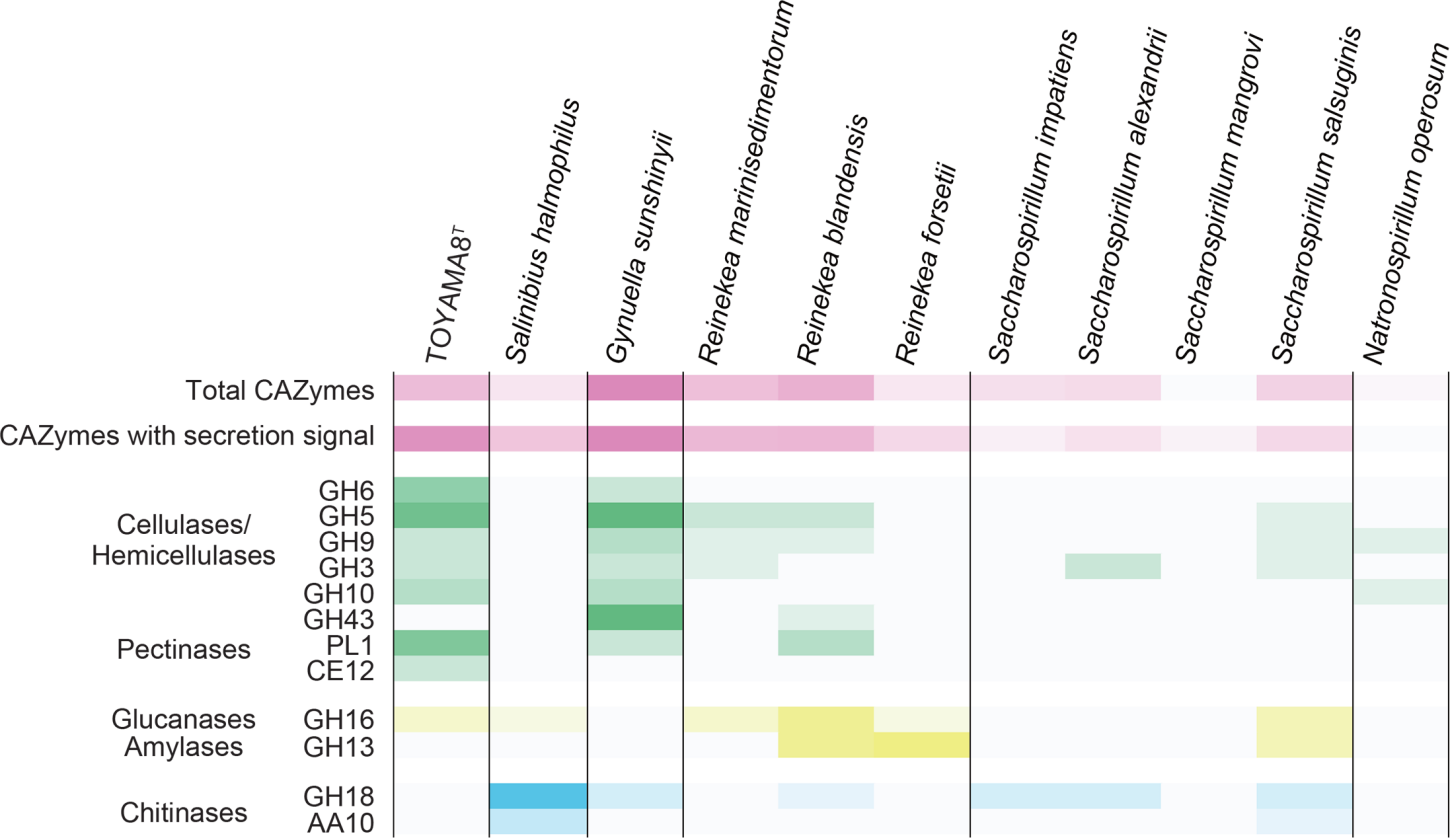
Putative CAZyme genes with secretion signal sequences found in strain TOYAMA8 and related species. The number of CAZyme genes found in the genome is shown as heatmaps with the maximum value in each category represented by the brightest colour (cellulases, hemicellulases and pectinases: green; glucanases and amylases: yellow; chitinases: blue). Abbreviations: GH, glycoside hydrolases; PL, polysaccharide lyases; CE, carbohydrate esterases; AA, auxiliary activities.

A search of the CAZyme database [27] revealed 113 CAZyme genes in the genome of the strain TOYAMA8. Notably, a large variety of putative extracellular cellulase, hemicellulase and pectinase genes are prominent features of this strain, which are advantageous for utilizing cellulolytic biomass as a nutrient source (Fig. 3). Although other species in the families *Gynuellaceae*, *Saccharospirillaceae* and *Natronospirillaceae* were also isolated from marine environments (Table 1), the presence of cellulase genes was uncommon, with only *G. sunshinyii* YC6258^T^ [28] speculated to have cellulolytic activity. This highlights the uniqueness of the screening technology using nanofibrous cellulose as a solid culture medium to isolate strains with characteristics different from those of micro-organisms found by conventional methods [2]. A detailed analysis of the genome and transcriptome of the strain TOYAMA8 can be found elsewhere [7].

We identified a metagenome-assembled genome (MAG) closely related to strain TOYAMA8 through GTDBtk analysis (Fig. S2), which assigned the strain to the GTDB taxonomy genus ‘DT-108’. Designated as ALOHA_DT_13 (Table 2), this MAG was derived from a deep sediment trap sample collected from the marine abyssalpelagic zone (4,000 m depth). The MAG ALOHA_DT_13 and strain TOYAMA8 shared an ANI of 74.7%, a dDDH value of 20.0% and a 16S rRNA identity of 98.8%, indicating that they belong to the same genus yet represent different species. To examine whether cellulolytic features are also present in the related species, we performed a CAZyme analysis of MAG ALOHA_DT_13. This MAG contained cellulase genes; however, the total number of CAZymes was lower than that in TOYAMA8 (Table 2). Furthermore, the specific cellulase variants differed between the two strains; whilst MAG lacks the hemicellulose family GH10, it possesses minor cellulase families, such as GH8 and GH45. Because genomic information on this genus is limited, and TOYAMA8 is currently the only isolated strain, the full extent of these characteristics remains unclear. Nevertheless, both organisms share two notable features; they originate from deep-sea environments and harbour cellulase genes.

**Table 2.** Comparison of genomic features between strain TOYAMA8 and MAG within the genus

Strain name	TOYAMA8	ALOHA_DT_135 (DT-108)
Sampling information	Isolated from deep-sea ascidian, Toyama Bay, Japan	MAG derived from deep trap particle (4,000 m), Hawaii
Genome accession number	AP023087	ASM2296898
Size (Mbp)	4.6	4.8
G+C content (mol%)	36.1	34.5
ANI (%)	74.7	
dDDH (%)	20.0	
16S rRNA sequence identity (%)	98.8	
Number of CAZymes*	113 (57)	81 (30)
Cellulase and hemicellulase families†	GH6 (5), GH5 (7), GH9 (2), GH10 (3)	GH6 (7), GH5 (6), GH9 (2), GH8 (1), GH45 (1)

*The numbers in parentheses indicate the number of genes containing secretory signals.

**†GH: glycoside hydrolase family; numbers in parentheses indicate the number of genes containing secretion signals.

## Physiology and chemotaxonomy

The effects of temperature, NaCl concentration and pH on cell growth were determined by examining the time course of the OD using a temperature gradient incubator or a compact rocking incubator with a TVS126MA or TVS062CA model bio-photorecorder (ADVANTEC). Growth was assessed at various temperatures (5, 9, 12, 14, 16, 18, 20, 22, 24, 26, 28 and 30 °C) in marine broth (MB) 2216 (Difco) containing 0.1% (w/v) cellobiose, and the optimal growth temperature was found to be 20 °C. The optimal pH and pH range for growth were determined in MB with 0.1% (w/v) cellobiose at 25 °C. The medium was adjusted to pH 6.0–9.5 (at intervals of 0.5 pH) using 10 mM MES (pH 6.0–6.5), 10 mM MOPS (pH 6.5–7.5), 10 mM HEPES (pH 7.5–8.5) and 10 mM Tris (pH 8.5–9.5). To test the effect of NaCl concentration on growth, NaCl was added to the basal medium at a final concentration of 0–60 g l^−1^.

Cell morphology was examined using an epifluorescence microscope (Olympus BX51F) equipped with a colour CCD camera system (Olympus DP72). Gram staining was performed using a BD Gram stain kit (Becton, Dickinson and Company) according to the manufacturer’s protocol. Transmission electron micrographs of the negatively stained cells were obtained as described by Zillig *et al*. [29]. Briefly, cells were collected during the mid-exponential growth phase at 20 °C in MB containing 0.1% (w/v) cellobiose and negatively stained with 2% (w/v) phosphotungstic acid for observation using a JEOL JEM-1210 electron microscope. Cells with flagella were also prepared, but for cultivation at 15 °C in a culture containing ASW, 1 mM ammonium sulphate, trace minerals, 0.1% casamino acids and cellulose gels, and observed with a Tecnai 20 electron microscope (FEI). For scanning electron microscopy (SEM), cells were allowed to adhere to a poly-ʟ-lysine-coated glass slide for 30 min at room temperature. The adhered cells were fixed in 2.5% (w/v) glutaraldehyde in maltose-yeast extract medium at 10 °C overnight and then washed in 0.1 M PBS (pH 7.4). Following this treatment, the cells were fixed in 2% (w/v) osmium tetroxide dissolved in PBS, dehydrated in a graded ethanol series and dried to a critical point in a JEOL JCPD-5 critical-point drier. The cells on the glass slides were coated with osmium using a Meiwa Shoji POC-3 osmium plasma coater and observed under a JEOL JSM-6700F field-emission scanning electron microscope operated at 5 kV.

For the analysis of isoprenoid quinones and cellular fatty acid methyl esters (FAMEs), strain TOYAMA8 cells were harvested from an exponential phase culture grown in MB with 0.1% (w/v) cellobiose. Detailed procedures for isoprenoid quinone and polar lipid analyses were performed as previously described [30]. FAME analysis of strain TOYAMA8 was performed on cells by saponification, methylation and extraction according to the Sherlock Microbial Identification System [31]. Dimethyl disulphide derivatives were added to the FAMEs during preparation to determine the double-bond positions of unsaturated fatty acids [32]. The fatty acid compositions were determined using a GC-MS system (JMS-Q1500GC, JEOL Ltd.) equipped with a DB-5MS column (30 m, J and W Scientific) under a helium flow of 1.5 ml min^−1^ and an oven temperature programme increasing from 120 to 260 °C (5 min) at 4 °C min^−1^. For the analysis of polar lipids, the strain TOYAMA8 was cultivated in liquid culture containing ASW, 1 mM ammonium sulphate, trace minerals, 0.2% cellobiose and 0.1% casamino acids at 20 °C for 3 days. Polar lipids were extracted from lyophilized cells (~50 mg) according to the procedures described by Minnikin *et al*. [33]. Polar lipids were separated by two-dimensional TLC and visualized by spraying with the appropriate detection reagents [34].

Oxidase activity was determined by spreading the cell pellets on oxidase test paper (Nissui Pharmaceutical). Catalase activity was determined based on O_2_-bubble production in a 3% (v/v) H_2_O_2_ solution. Agarase, amylase, cellulase, gelatinase, lipase (tri-n-butyrin) and protease activities were tested on MA using cellobiose plates at a substrate concentration of 1% (w/v). The DNase activity was assessed using DNase test agar (Difco). Other physiological and biochemical characteristics were examined using API 20NE, API ZYM and API 50CH test strips (bioMérieux) according to the manufacturer’s instructions, except for the use of ASW.

**Fig. 4.**
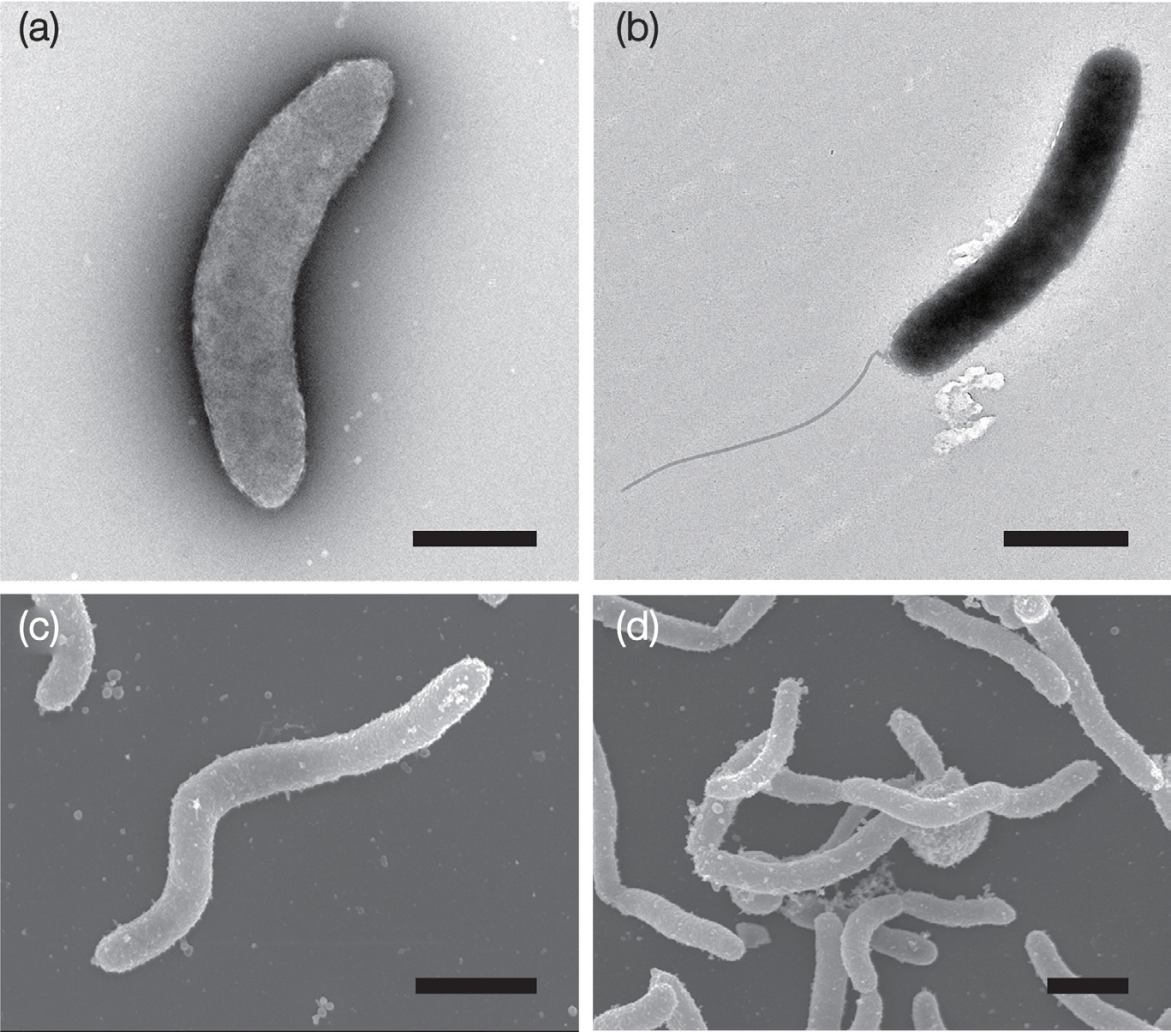
Transmission electron microscopy (**a, b**) and SEM (**c, d**) images of strain TOYAMA8. Cells were grown on cultures containing cellobiose (a, c and d) or cellulose (**b**). Scale bars: 1 µm.

**Fig. 5.**
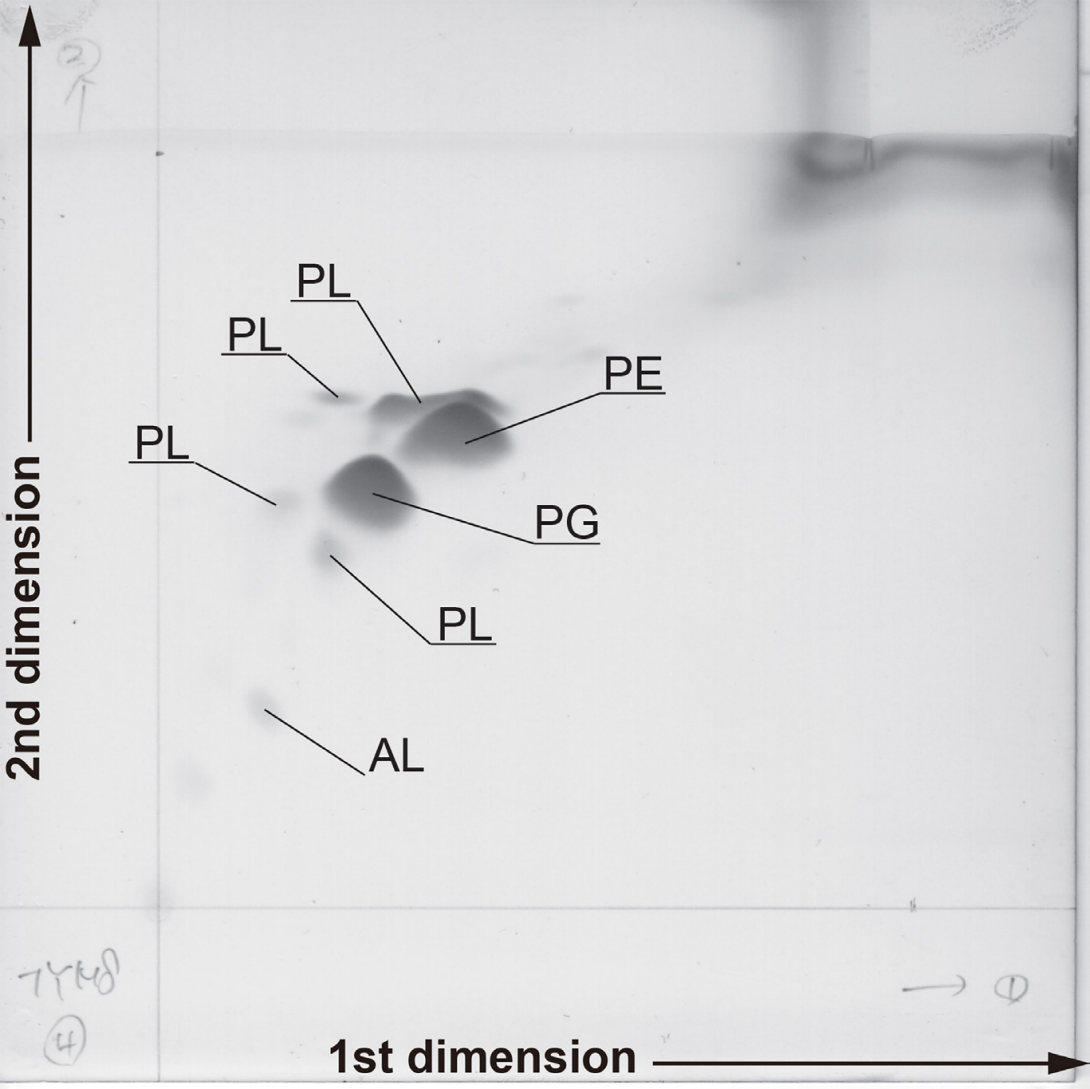
Polar lipid profiles of strain TOYAMA8 after two-dimensional TLC. PG, phosphatidylglycerol; PE, phosphatidylethanolamine; PLs, unidentified phospholipids; AL, aminolipids.

Cells of strain TOYAMA8 were Gram-stain-negative, obligately aerobic, curved rods or spirilla (Fig. 4), and coccoid bodies were observed in older cultures. Motile cells with monopolar flagella were observed when the strain was grown on cellulose (Fig. 3b); however, most cells were non-motile, and motile cells were rarely observed when grown on cellobiose. The presence of flagella was species-dependent in the related genera (Table 1). Strain TOYAMA8^T^ showed alkaliphilic growth within the pH range 7.5–9.0. The growth temperature range (5–26 °C, no growth was observed above 28 °C) and optimum temperature (20 °C) of strain TOYAMA8 were lower than in the other related strains (Table 1). TOYAMA8 grew on glucose, cellobiose or cellulose as its sole carbon source; however, many other sugars and aa did not support growth, as listed in the species description. Its specialization for cellulose as an energy source is relatively unique compared to related species that also grow on other sugars (Table 1). Additionally, cellulose degradation ability was reported for *G. sunshinyii* YC6258^T^ [28], and carboxymethyl cellulose was degraded by a few species in the genera *Reinekea* [35,36] and *Saccharospirillum* [37]; however, this was not evidence of cellulose hydrolysis because carboxymethyl cellulose can be degraded by *β*-glucanases. The major cellular fatty acids were C_18 : 1_ ω7 (64.6%), C_14 : 0_ (17.8%), C_16 : 0_ (8.0%) and C_16 : 1_ ω7 (5.8%). In contrast, four related strains, *S. halmophilus* WDS2A16A^T^, *G. sunshinyii* YC6258^T^, *Saccharospirillum impatiens* EL-105^T^ and *Reinekea marinisedimentorum* DSM 15388^T^, contained C_16 : 0_ or C_16 : 1_ as the second largest proportion after C_18 : 1_ ω7 (Table 3). The major polar lipids are phosphatidylglycerol (PG), phosphatidylethanolamine (PE), unidentified phospholipids (PLs) and aminolipids (ALs) (Fig. 5). The major respiratory lipoquinone was Q-9, whereas the respiratory lipoquinone of most other strains in related genera was Q-8 (Table 1). The following antibiotics showed sensitivity at 100 µg ml^−1^: ampicillin, bacitracin, chloramphenicol, gentamycin, kanamycin, neomycin, penicillin, streptomycin, tetracycline and vancomycin, whereas rifampicin showed sensitivity at 20 µg ml^−1^. Other physiological and biochemical characteristics are listed in the species description section and Table 1.

**Table 3.** Fatty acid contents (%) of strain TOYAMA8 and the related species Strains: 1. TOYAMA8^T^; 2. *S. halmophilus* WDS2A16A^T^; 3. *G. sunshinyii* YC6258^T^; 4. *S. impatiens* EL-105^T^; and 5. *R. marinisedimentorum* DSM 15388^T^. Collated using data from previous studies (Ling *et al*. [25], Chung *et al*. [28], Labrenz *et al*. [40] and Romanenko *et al*. [36]). –, not detected; tr<1%.

Fatty acid	1	2	3	4	5§
Straight chain					
C_11 : 0_	–	–	tr	–	–
C_12 : 0_	–	tr	–	–	–
C_14 : 0_	**17.8**	1.7	–	–	2.0
Methyl-C_14 : 0_	–	–	tr	–	–
C_15 : 0_	tr	7.8	–	–	4.1
C_16 : 0_	8.0	**27.8**	**27.3**	20†	31.6
C_17 : 0_	–	3.4	1.1	–	5.9
C_18 : 0_	tr	1.0	1.4	–	tr
Blanched					
Anteiso- C_15 : 0_	–	tr	–	–	–
Unsaturated					
C_14 : 1_ ω7	1.6	–	–	–	–
C_16 : 1_ ω7	5.8	–	–	23†	26.7
C_17 : 1_ ω6c	–	2.4	tr	–	1.9
C_17 : 1_ ω8c	–	tr	tr	–	1.3
C_18 : 1_ ω7	**64.6**	**41.8**	**32.2**	54†	19.0
C_19 : 1_	–	–	–	2†	–
Hydroxy					
C_10 : 0_ 3-OH	tr	–	5.5	12‡	–
C_10 : 1_ 3-OH	–	–	–	4‡	–
C_12 : 0_ 3-OH	–	–	1.2	7‡	–
C_14 : 0_ 3-OH				31‡	2.4
C_14 : 1_ 3-OH				44‡	–
C_16 : 0_ 3-OH	–	–	1.3	–	–
Summed features*		–			
1	–	–	tr	–	–
2	–	–	10.0	–	–
3	–	7.3	**16.5**	–	–
7	–	4.7	–	–	–

*Summed feature 1 contains iso-C_15 : 0_ h and/or C_13 : 0_ 3-OH, summed feature 2 contains C_12 : 0_ aldehyde, summed feature 3 contains C_16 : 1_ ω7c and/or C_16 : 1_ ω6c, and summed feature 7 contains C_19 : 1_ ω6c and/or unknown 18.846.

†Numbers refer to the percentage of an acid relative to the total non-polar fatty acids.

‡Numbers refer to the percentage of an acid relative to the total hydroxy fatty acids.

§In addition to those listed in the table, the following fatty acids were detected: C_16 : 0_ N alcohol (1.2%), C_17 : 0_ 10-methyl (<1%), and C_12 : 0_ ALDE (<1%).

Based on the phylogenetic, chemotaxonomic and other taxonomic data, strain TOYAMA8 represents a novel species of a new genus in the class *Gammaproteobacteria*, for which the name *Marinicellulosiphila megalodicopiae* gen. nov., sp. nov., has been proposed.

## Description of *Marinicellulosiphila* gen. nov.

*Marinicellulosiphila* (Ma.ri.ni.cel.lu.lo.si'phi.la. L. fem. adj. marina of the sea, marine; N. L. neut. n. *cellulosum*, cellulose; N.L. fem. adj. suff. *-phila*, loving; N.L. fem. n. *Marinicellulosiphila*, marine, cellulose-loving).

Cells are Gram-negative, aerobic, heterotrophic bacteria that grow with cellulose as the sole carbon source. Na^+^ ions are essential for cell growth. Oxidase test is positive and catalase test is negative. The major cellular fatty acids are C_18 : 1_ ω7 and C_14 : 0_. The major polar lipids include PG, PE, PLs and ALs. A major respiratory lipoquinone is Q-9. The genus is phylogenetically affiliated with the class *Gammaproteobacteria*. The type strain is *M. megalodicopiae*.

## Description of *Marinicellulosiphila megalodicopiae* sp. nov.

*Marinicellulosiphila megalodicopiae* (me.ga.lo.di.co’pi.ae. N.L. gen. n. *megalodicopiae*, of *Megalodicopia,* adhering to *Megalodicopia hians*)

The following characteristics are displayed in addition to the properties of the genus description. Cells are curved rods or spirilla usually 0.3–0.5 µm wide and 1.8–5.4 µm long. Cells are motile with monopolar flagella. Coccoid bodies are formed in older cultures. Optimal growth occurs at 20 °C (range, 5–26 °C), whilst no growth occurs above 28 °C. Growth occurs at an optimum of ~1.5–2.5% (w/v) NaCl (range 0.5–5.5%); no growth occurs at 0% (w/v) and above 6% (w/v) NaCl. The growth pH range is between pH 7.5 and 9.0; no growth occurs below pH 7.0 and above pH 9.5. No activity was detected for amylase, gelatinase, lipase (tri-n-butyrin), protease (casein) and DNase. Cells grow using ᴅ-glucose, ᴅ-cellobiose, or cellulose as the sole carbon and energy sources. Growth is not achieved with the following substrates as carbon and energy sources: ᴅ-arabinose, ʟ-arabinose, ᴅ-fructose, ᴅ-galactose, glycerol, myo-inositol, ᴅ-lactose, maltose, ᴅ-mannose, ᴅ-mannitol, ʟ-rhamnose, ᴅ-raffinose, ribitol, ᴅ-ribose, ᴅ-sorbitol, sorbose, sucrose, ᴅ-trehalose, xylitol, methanol, ethanol, 1-propanol, 2-propanol, cyclopropanol, 1-butanol, 2-butanol, methylamine, dimethylamine, trimethylamine, acetate, butyrate, citrate, crotonate, formate, fumarate, lactate, malate, propionate, pyruvate (so far, 10 mM), ʟ-alanine, ʟ-arginine, ʟ-asparagine, ᴅ-aspartic acid, ʟ-cysteine, ʟ-cystine, ᴅ-glutamic acid, ʟ-glutamine, ʟ-glycine, ʟ-histidine, ʟ-isoleucine, ʟ-leucine, ʟ-lysine, ᴅʟ-methionine, ʟ-norvaline, ʟ-ornithine, ʟ-phenylalanine, ʟ-proline, ʟ-serine, ʟ-threonine, ʟ-tryptophan, ʟ-tyrosine, ʟ-valine (5 mM), yeast extract, peptone, casamino acids and beef extract (0.1%). The API ZYM test was positive for alkaline phosphomonoesterase, esterase (C4), esterase lipase (C8), leucine arylamidase, valine arylamidase, *β*-glucosidase and *N*-acetyl-*β*-glucosaminidase and negative for lipase (C14), cystine arylamidase, trypsin, *α*-chymotrypsin, acid phosphomonoesterase, naphthol-AS-BI-phosphoamidase, *α*-galactosidase, *β*-galactosidase, *β*-glucuronidase, *α*-glucosidase, *α*-mannosidase and *α*-fucosidase. The API 20NE test was positive for reduction of nitrates to nitrites, urease, hydrolysis of aesculin and *β*-galactosidase and negative for indole production, glucose fermentation, arginine dihydrolase and hydrolysis of gelatin.

The type strain TOYAMA8^T^ (=JCM 31119^T^=DSM 114864^T^) was isolated from a slurry-adhered epiphytic site of *M. hians* in Toyama Bay, Japan. The GenBank/EMBL/DDBJ accession numbers for the complete genome of strain TOYAMA8^T^ and 16S rRNA gene sequences are AP023087 and LC833869, respectively. The G+C content of the DNA of the type strain is 36.1 mol%, and its genome size is 4.65 Mbp.

## supplementary material

10.1099/ijsem.0.006742 Uncited Supplementary Material 1.

## Abbreviations

AAI: average amino acid identity
ALs: aminolipids
ANI: average nucleotide identity
ASW: artificial seawater
CAZyme: carbohydrate-active enzyme
FAMEs: fatty acid methyl esters
GTDB: Genome Taxonomy Database
MA: marine agar
MAG: metagenome-assembled genome
MB: marine broth
PE: phosphatidylethanolamine
PG: phosphatidylglycerol
PLs: unidentified phospholipids
POCP: percentage of conserved proteins
SEM: scanning electron microscopy
