# Supplementary material for: Marinicellulosiphila megalodicopiae gen. nov., sp. nov., a deep-sea alkaliphilic cellulolytic bacterium isolated from an endemic ascidian Megalodicopia hians
Source: Int J Syst Evol Microbiol. 2025 Apr 2;75(4):006742. doi: 10.1099/ijsem.0.006742 (PMC11966569; doi:10.1099/ijsem.0.006742)
Supplement: Uncited Supplementary Material 1. [file ijsem-75-06742-s001.pdf]

Supplementary data

Marinicellulosiphila megalodicopiae gen. nov., sp. nov., a deep-sea alkaliphilic cellulolytic bacterium isolated from an endemic ascidian *Megalodicopia hians*

Mikako Tachioka[1], Masayuki Miyazaki[2], Mikiko Tsudome[1], Miwako Tsuda[3], Kohsuke Uchimura[1], Yoshihiro Takaki[1] & Shigeru Deguchi[2,*]

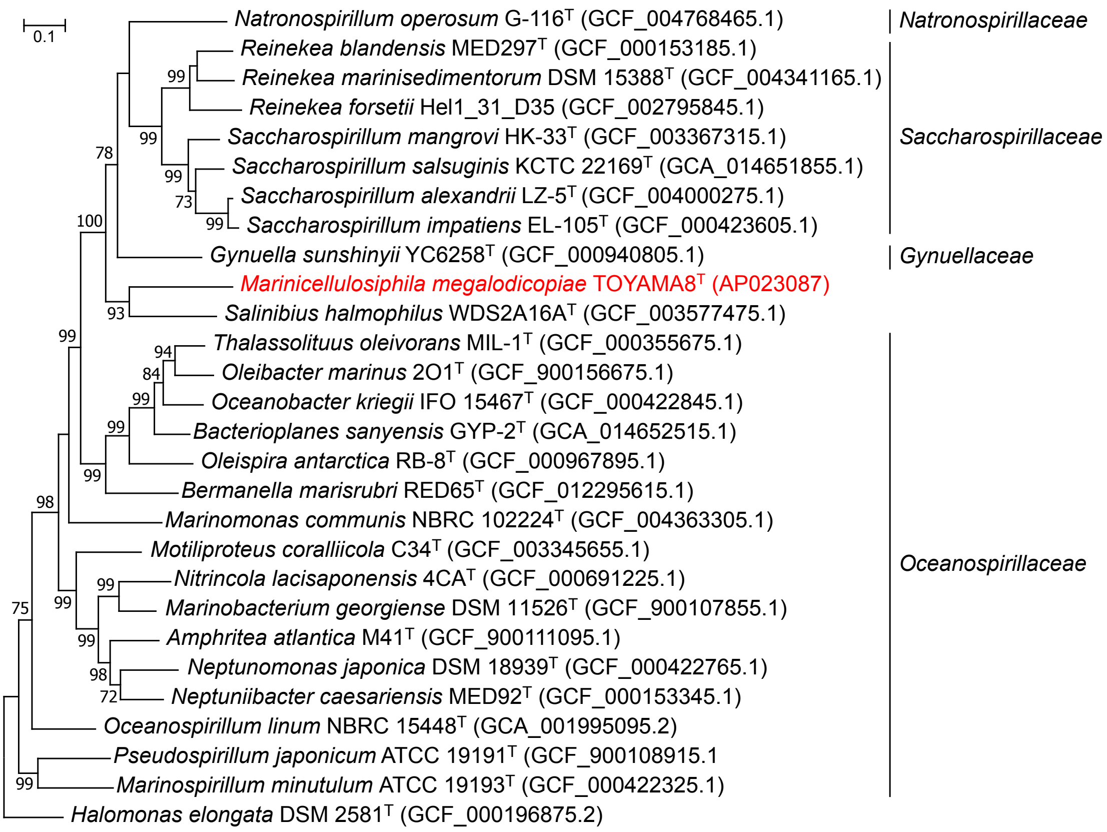

Supplementary Fig S1. Maximum-likelihood tree based on 120 concatenated single copy marker proteins of the strain TOYAMA8 and related genera in the family *Saccharospirillaceae*, *Gynuellaceae*, *Natronospirillaceae* and *Oceanospirillaceae*. Bootstrap values (≧70 %, 300 replications) are shown at nodes. Bar, 0.1 substitutions per nucleotide position. The tree was rooted using *Halomonas elongata* DSM 2581^T as the outgroup.

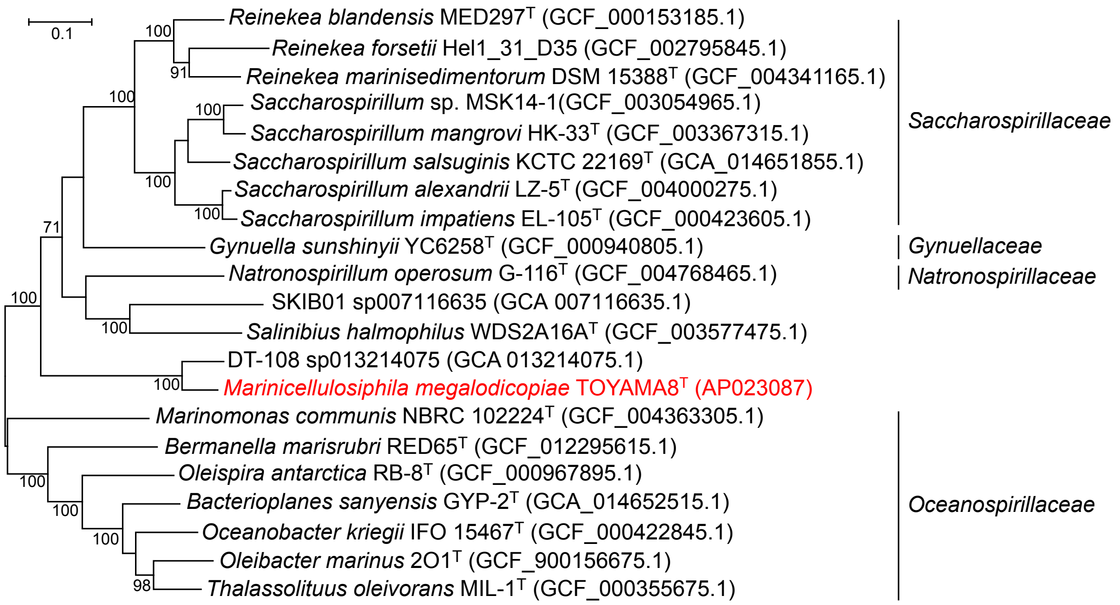

Supplementary Fig S2. Maximum-likelihood tree based on 120 concatenated single copy marker proteins of the strain TOYAMA8 and related species including environmental samples in the family *Saccharospirillaceae*, *Gynuellaceae*, and *Natronospirillaceae*. Bootstrap values (≧70 %, 300 replications) are shown at nodes. Bar, 0.1 substitutions per nucleotide position. A total 3,624 positions were used to calculate the tree. The tree was rooted using *Oceanospirillaceae* group.
